# Molecular and Evolutionary Analysis of RNA–Protein Interactions in Telomerase Regulation

**DOI:** 10.3390/ncrna10030036

**Published:** 2024-06-18

**Authors:** Justin A. Davis, Kausik Chakrabarti

**Affiliations:** Department of Biological Sciences, University of North Carolina at Charlotte, Charlotte, NC 28223, USA; jdavi364@charlotte.edu

**Keywords:** telomerase, telomeres, telomerase RNA, telomerase reverse transcriptase, RNA-binding proteins, RNA chaperones, interactomes, parasites, *Trypanosoma brucei*

## Abstract

Telomerase is an enzyme involved in the maintenance of telomeres. Telomere shortening due to the end-replication problem is a threat to the genome integrity of all eukaryotes. Telomerase inside cells depends on a myriad of protein–protein and RNA–protein interactions to properly assemble and regulate the function of the telomerase holoenzyme. These interactions are well studied in model eukaryotes, like humans, yeast, and the ciliated protozoan known as *Tetrahymena thermophila*. Emerging evidence also suggests that deep-branching eukaryotes, such as the parasitic protist *Trypanosoma brucei* require conserved and novel RNA-binding proteins for the assembly and function of their telomerase. In this review, we will discuss telomerase regulatory pathways in the context of telomerase-interacting proteins, with special attention paid to RNA-binding proteins. We will discuss these interactors on an evolutionary scale, from parasitic protists to humans, to provide a broader perspective on the extensive role that protein–protein and RNA–protein interactions play in regulating telomerase activity in eukaryotes.

## 1. Introduction

The regulation of telomere elongation in eukaryotes entails unique and extensive protein–nucleic acid interactions. Telomeres are DNA–protein complexes found at the ends of eukaryotic linear chromosomes. These structures provide a protective cap at the ends of telomeres to preserve the integrity of an organism’s genome [1]. In mammalian cells, this telomeric DNA is also associated with a variety of DNA-binding proteins that form the Shelterin complex [2]. The protein components of the mammalian Shelterin complex, include telomere repeat binding factor 1 (TRF1), telomere repeat-binding factor 2 (TRF2), the protection of telomere 1 (POT1), repressor/activator protein 1 (RAP1), TRF1- and TRF2-interacting nuclear protein 2 (TIN2), and TPP1. Similar telomeric capping complexes exist in other organisms like yeast, whose “telosomes” mainly consist of a yeast homolog of RAP1 [3]. These proteins bind to and shelter the double- and single-stranded regions of telomeric DNA. The Shelterin complex caps telomeres and prevents them from being degraded and recognized as a form of DNA damage inside the cells. Conventional DNA polymerases inside the cells are unable to replicate DNA to their ends because of the end-replication problem [4]. Progressive telomere shortening leads to a compromise of genome integrity with many deleterious outcomes. These include the following: DNA damage, cellular senescence, and apoptosis [5]. The telomeric DNA is composed of both single-stranded and double-stranded DNA regions. However, cells have developed a solution to this problem called telomerase. Telomerase is a ribonucleoprotein that extends telomeres to maintain their length. Telomerase consists of two main components: the telomerase reverse transcriptase (TERT) protein that extends telomeres using its reverse transcriptase activity and the telomerase RNA (TR) that provides the template for telomere repeat synthesis [6].

The TERT protein and the TR are the minimum components necessary to reconstitute telomerase activity in vitro [7,8]. In vivo, there are numerous accessory factors that interact with the TERT and the TR to form the complete telomerase holoenzyme. The TERT subunit of telomerase is evolutionarily conserved in diverse organisms from protozoa and fungi to mammals. The TR itself is a long non-coding RNA that forms a large structural scaffold upon which many accessory protein factors can bind. These interactions are not only required for the processing and maturation of the TR, but also for the proper assembly and biogenesis of the telomerase complex [9]. These pathways, and the many protein factors that function in them, have been well studied and identified in model eukaryotes. Less is known about these pathways and interacting proteins in protozoans, including clinically relevant human parasites. In this review, we will discuss what is known about RNA-interacting proteins in telomerase regulation and highlight recent studies carried out in eukaryotes of early origin, as well as in higher eukaryotes, which are extensively studied in telomerase biology to highlight the conserved and unique mechanisms of telomerase action and regulation across eukaryotes.

## 2. Protein Interactors of the Telomerase Ribonucleoprotein

### 2.1. Core Telomerase-Interacting Proteins across Eukaryotes

The TERT protein contains four important functional domains. These domains are the telomerase essential N-terminal (TEN) domain, the telomerase RNA-binding domain (TRBD), the reverse transcriptase (RT) domain, and the C-terminal extension (CTE) domain. The TR molecule, like the TERT protein, also contains important structural domains that play key roles in telomerase activity and regulation. These important functional domains include the template domain, the template boundary element, the pseudoknot, and the CR4/5 domain [10]. Several of these important functional domains also play key roles in TERT-TR interaction and binding. The TEN and the TRBD domain of the TERT were found to bind to the TR CR4/5 domain and the pseudoknot to promote telomerase assembly [11,12,13]. The template boundary element within the TR molecule has also been shown to be a TERT binding site, which may serve to properly position the template boundary element during telomere synthesis [14]. The CR4/5, pseudoknot, and template boundary element are highly conserved domains within the TR molecule. These domains are present in diverse eukaryotic lineages and are present in vertebrates, fission yeast, and filamentous fungi [15]. This conservation across diverse eukaryotic lineages highlights the important role that these domains play in the facilitation of TERT-TR binding and telomerase assembly. In vivo, there are many additional accessory proteins that bind to the TERT protein and the TR to collectively form the complete telomerase holoenzyme. In addition to the binding of the TERT protein, several structural domains within the TR molecule act as hot spots for RNA-binding proteins. Well-studied examples of this include the H/ACA box domain in vertebrate TR and the CR4/5 domain [16]. These accessory protein interactions are critical for the proper functioning and regulation of the telomerase enzyme within living systems. Because of their importance, these interacting proteins of telomerase have been identified using a variety of approaches, including large-scale proteomics experiments using mass spectrometry and structural biology. In this section, we will comprehensively discuss the unique proteins identified in telomerase RNA–protein complexes across eukaryotes, and the role of RNA-binding proteins will be discussed in more detail in the next section.

The origin of TERT proteins is likely linked to RNA-dependent RNA polymerases that use the same set of reverse transcriptase (RT) motifs relating to non-LTR retrotransposons [17]. In particular, Penelope-like elements (PLEs) and retrotransposons RTs show a similar sequence to the TERT protein [18]. The TR is also proposed to derive from sequences associated with Group II introns that are associated with a Group II reverse transcriptase [18]. In this model, the reverse transcriptase and the future TR would be encoded by two different self-splicing Group II introns. Over the course of evolutionary time, these two separate Group II introns have developed into their own independent genes [18]. During evolution, TERT has evolved as an essential factor for protecting telomeres by interacting with several accessory proteins directly or via its integral RNA molecule. Interacting proteins of human TERT (hTERT) have been identified using proteomics and structural biology techniques. Initial mass-spectrometry-based interactome studies of a catalytically active human TERT complex only identified the H/ACA-box-binding protein dyskerin as a part of the active telomerase complex in human cells [19]. Subsequent mass spectrometry experiments of both catalytically active and inactive human telomerase complexes identified a more comprehensive hTERT interactome [20]. The interactors that were identified included core H/ACA box small nucleolar-binding proteins (snoRNPs), like dyskerin, NHP2, and NOP10 (Table 1). Several proteins were also identified from the hnRNP and Sm class of RNA-binding proteins [20]. Follow-up studies looking at both the hTERT and dyskerin interactomes identified many of the same interactors and additional factors, such as GAR1 and TCAB1 [21]. The cryo-EM structure of the human telomerase holoenzyme has been solved in conjunction with telomeric DNA [22,23]. This high-resolution structure demonstrated that NHP2, NOP10, TCAB1, GAR1, and dyskerin are important components of the telomerase holoenzyme when bound to its substrate.

The cryo-EM structure of the yeast telomerase holoenzyme remains to be solved. However, key interacting proteins of telomerase in *Saccharomyces cerevisiae* (*S. cerevisiae*) have been identified using biochemical and mass-spectrometry-based approaches. The interacting proteins identified are as follows: Est1, Est3, Pop1, Pop6, Pop7, Ku 70/80, Smd2, Smb1, and Smd3 (Table 1) [34]. These protein interactors include proteins involved in the following: telomere replication, DNA repair, and RNA processing and stability. Many of these identified proteins are a part of the telomerase holoenzyme in yeast and are required for the enzymatic activity and the proper biogenesis of the telomerase complex.

Another higher eukaryotic system in which TERT-interacting proteins have been identified is plants. The TR has been identified in the model plant *Arabidopsis* and its secondary structure has been determined [35,36]. The identification of telomerase-interacting proteins is not as extensive in plants, but several novel and conserved interactions with *Arabidopsis thaliana* (*A. thaliana*) TERT (*At*TERT) have been identified [37]. These interactions include a direct association between *At*TERT and TRB proteins [38]. These TRB proteins in *Arabidopsis* have a Myb domain at their N-terminus and a central histone-like domain. These proteins appear to be functional homologs of mammalian TRF in plants. There are also several other TRF-like proteins identified in *Arabidopsis* that have a Myb domain at their C-terminus. The TRB proteins that contain a Myb domain at their N-terminus may also play a role in recruiting *At*TERT to telomeres because they were shown to interact with *At*TERT [38]. In *Arabidopsis*, it is possible that TRB family proteins are components of a telomere-binding complex that plays roles in telomerase recruitment, analogous to TPP1 that is observed in the mammalian shelterin complex [38,39,40]. Another telomeric protein that it was found to be associated with was POT1. *A. thaliana’s* genome encodes for two paralogs of POT1. These proteins are POT1a and POT1b [41]. Interestingly, POT1a was the only one found to interact with *At*TERT [37,42]. A notable conserved interaction in *A. thaliana* telomerase is the association with the dyskerin protein [42,43]. The interaction with dyskerin in *A. thaliana* is also required for proper telomerase activity like what is observed in humans. This interaction with the dyskerin protein is unusual as the TR gene in plants is transcribed by RNA POL III and lacks an H/ACA box domain [36]. This demonstrates that telomerase in plants is associated with conserved interacting proteins from vertebrates but retains some ciliate characteristics [36,42]. 

Proteins that form the complete telomerase holoenzyme in the free-living ciliate *Tetrahymena thermophila* (*T. thermophila*) have been identified using a combination of mass spectrometry and structural biology approaches. Initial interactome studies of the *T. thermophila* telomerase complex identified p65, p82, p75, p45, p19, and p50 as part of the telomerase holoenzyme [44]. The proteins p75, p19, and p45 form a subcomplex, while p65 directly interacts with the *T. thermophila* telomerase RNA (*Tt*TR). The structure of the *T. thermophila* telomerase holoenzyme has been solved by cryo-EM [27]. This structure revealed previously unknown interactions that form the complete telomerase holoenzyme in *T. thermophila*. The complete holoenzyme components of the *T. thermophila* holoenzyme are as follows: p65, p75, p45, p19, p50, Teb1, Teb2, and Teb3. Teb1-Teb2-Teb3 (Table 1) are paralogs of the mammalian single-stranded DNA-binding proteins known as RPA. Teb1, Teb2, and Teb3 form their own subcomplex and have been shown to be important for stabilizing the telomerase holoenzyme at telomeres [27]. 

There are unique and conserved interactions of telomerase across eukaryotes. The TERT protein is well conserved across a variety of eukaryotic phyla from humans to parasitic protists [10]. The heterogeneity of the protein interactions does not solely arise from differences in the TERT protein, but also from differences in the TR. The TR differs in both size, structure, and sequence composition across eukaryotes. The structures of the TR have been identified in a variety of eukaryotes, including parasitic protozoa, like *T. brucei* [45,46]. The telomerase-interacting proteins have not been well studied in relation to parasitic protozoa. A recent interactome study of *T. brucei* TERT (*Tb*TERT) carried out by our group identified conserved and novel protein interactions in the *T. brucei* telomerase complex [32]. Conserved interactions included an association with a variety of molecular chaperones, including the mitochondrial chaperone HSP60 [47,48]. Another notably conserved interaction was with a *T. brucei* la protein (Tb927.10.2370), which is a potential homolog of p65 from *Tetrahymena* [32]. The TR in humans contains an H/ACA snoRNA domain that is replaced by a C/D box domain in *T. brucei* [33] TR. Novel interactions included an association with C/D-box-binding proteins, such as NOP56, NOP58, and fibrillarin (NOP1). These findings support earlier studies [33] and highlight unique mechanisms of telomerase regulation that may exist in *T. brucei*. 

In addition, the catalytic component of telomerase RNP, the TERT protein, was also identified in other protists, such as in *Paramecium* sp., *Plasmodium falciparum*, *Leishmania major*, *Paramecium caudatum*, *Giardia intestinalis*, *Leishmanis sp* [49,50,51,52,53], and *Dictyostelium discoideum* [54]. However, their species-specific unique telomerase-interacting partners are yet to be identified and characterized at the molecular level. 

### 2.2. Interactions of Telomerase with RNA-binding Proteins and RNA Chaperones

The complete telomerase holoenzyme in eukaryotes is composed of a variety of accessory proteins and could be dynamic in nature. A very important class of interacting proteins is RNA-binding proteins. These proteins bind to and interact with the TR molecule, which provides a large structural scaffold for the telomerase holoenzyme complex. In this section, we will highlight and discuss the role of RNA-binding proteins and RNA chaperones in telomerase regulation across representative eukaryotic taxa.

RNA chaperones represent an important class of RNA-binding proteins that participate in telomerase regulation. RNA chaperones are RNA-binding proteins that can interact with RNA molecules to stabilize them and ensure they fold into a functional conformation [55]. An important RNA chaperone that interacts with hTR is TCAB1. TCAB1 binds with RNA molecules through a four-nucleotide CAB box motif and targets them to Cajal bodies [56]. TCAB1 is a Cajal-body-associated protein that is required for human telomerase activity via an RNA activity switch. TCAB1 exerts its RNA chaperone activity by regulating the folding of the CR4/5 domain, an important structural domain required for telomerase activity, into an active conformation in the telomerase RNA molecule [24]. Once TCAB1 is bound, and the CR4/5 domain properly folds, telomerase activity can occur. In addition to regulating telomerase catalytic activity, TCAB1 interaction is also required for telomerase accumulation in the Cajal bodies and the proper recruitment of telomerase to telomeres inside the cell [57]. 

The telomerase holoenzyme in *S. cerevisiae* interacts with a diverse set of RNA-binding proteins, including the Pop1, Pop6, Pop7, Smd2, Smb1, and Smd3 (Table 1), which engage RNA through an RNA recognition motif (RRM) and charge–charge interactions. Pop1, Pop6, and Pop7 are RNA-binding proteins canonically associated with RNase P/MRP molecules. These proteins are required for telomerase activity by stabilizing the TR molecule and Est1-Est3 interactions in the telomerase holoenzyme [26]. Smd2, Smb1, and Smd3 are small nuclear proteins that are canonically involved in pre-mRNA splicing. These proteins collectively bind to the 3′ end of *S. cerevisiae* TR to form the Sm7 complex, which binds to a unique Sm sequence motif within the yeast TR. The Sm7 complex is required for telomerase activity and stability, as well as *S. cerevisiae* TR processing [58]. 

RNA-binding proteins that are associated with the *T. thermophila* telomerase holoenzyme are as follows: p65, p75, p45, p19, and p50. These RNA-binding proteins are required to promote the proper stability, assembly, and activity of the *T. thermophila* telomerase holoenzyme [59]. A well-studied RNA chaperone in this holoenzyme is p65. This protein belongs to the La family class of RNA-binding proteins that are well conserved across eukaryotes [60]. The chaperone activity of p65 has been well studied using a combination of RNA selective 2′ hydroxyl acylation analyzed with primer extension (RNA-SHAPE) probing and structural biology techniques. The interactions of p65 have been shown to be required for the proper folding of the *Tetrahymena thermophila* telomerase RNA (*Tt*TR) and the assembly of the telomerase holoenzyme [28,29,30]. Specifically, p65 binds to the *Tt*TR and orients the SL4 stem helix, fixing it in place to facilitate interactions with the TERT protein. The interaction with p65 is also proposed to stabilize the pseudoknot structure in the *Tt*TR to allow interaction with the TERT protein for proper telomerase assembly and activity [30].

RNA-binding proteins play critical roles in the regulation of telomerase RNPs across a diverse set of eukaryotic species. Recent studies have also highlighted the importance of these diverse sets of RNA-binding proteins in parasitic protozoa, like *T. brucei*. *T. brucei* telomerase has been shown to be associated with core C/D box snoRNA-binding proteins, like NOP58, NOP56, and fibrillarin (NOP1). The exact role for these proteins in *T. brucei* is unknown, but they may be required for proper TR stability and telomerase assembly, as expected from studies with a hTR H/ACA domain [61,62]. The TR of a closely related parasite, *Leishmania*, also contains a C/D box domain [63]. The conservation of a C/D box domain in these parasites may represent the presence of a conserved mechanism in kinetoplastid parasites for telomerase regulation, mediated by C/D snoRNPs rather than H/ACA snoRNPs. 

A collection of several of these RNAs and proteins from model eukaryotes, as well as information on other species, i.e., TR, TERT, and accessory proteins (and their sequence, secondary and tertiary structure, etc.), is available in the Telomerase database (https://telomerase.asu.edu/, accessed on 11 April 2024) [64]. 

### 2.3. Technologies Used to Identify Global Interactors of Telomerase Holoenzymes

Identifying the interacting proteins of a protein of interest is critical in understanding the function of a protein inside the cell and how it is regulated. The global set of interactors of a protein of interest is called the “interactome”. Mass-spectrometry-based proteomics is a rapidly developing technology that is routinely used to study global protein interactions. These proteomics technologies have been applied to various eukaryotic organisms to study the global interactions of telomerase. In this section, we will detail successful applications of mass-spectrometry-based proteomics to gain new insight into telomerase interactors.

Affinity purification–mass spectrometry (AP-MS) is routinely used to identify interacting proteins of a specific protein of interest. Mass spectrometry has been applied to identify interacting proteins of the human telomerase holoenzyme (Table 2). The initial interactome profiling of the hTERT protein only identified dyskerin as an interacting protein [19]. This was likely due to the stringent purification methods used to selectively purify active telomerase complexes. Subsequent AP-MS profiling experiments revealed a more extensive list of interacting proteins, including core H/ACA-box-binding proteins and other unique RNA-binding proteins discussed earlier [20]. AP-MS techniques have also been successfully utilized to study the global interactors of telomerase across diverse eukaryotes, including *T. thermophila*, yeast, and *T. brucei* [25,26,27,31,32].

Typical AP-MS experiments are good at identifying stable interactions but often miss less stable and transient protein interactions. Improvements have been made in traditional AP-MS experimental methods in the form of proximity-labeling techniques, where proximity-labeling enzymes can be specifically targeted to protein complexes and label proteins in close proximity to a protein of interest [65]. These labeled proteins can then be pulled down using affinity purification techniques. Proximity-labeling techniques have recently been used to identify novel interactors of the hTERT protein [66]. This identified the ALKBH5 m6A demethylase as an interactor of the hTR molecule. This showed, for the first time, that post-transcriptional modifications, like m6A, play a role in regulating telomerase function. This approach, using an engineered ascorbate peroxidase (APEX) labelling strategy, targets the TR rather than the protein component TERT. A previous study also took an RNA-centric approach to identify the RNA interactome of hTR [67]. This approach was completed using antisense oligonucleotides to pull down the hTR molecule, which was followed by RNA sequencing to identify novel RNA molecules that are associated with the hTR molecule. 

**Table 2:** Methods used to identify the global protein interactors of telomerase.

Species	Technology	References
Humans	AP-MS; APEX labeling	[19,20,66]
*T. thermophila*	AP-MS	[27,31]
*S. cerevisiae*	AP-MS	[25,26]
*T. brucei*	AP-MS	[32]

## 3. RNA-Binding Proteins in the Maturation and Processing Stages of the Telomerase RNA

### 3.1. Telomerase RNA Processing and Biogenesis

The TR molecule is one of the most divergent components of the telomerase holoenzyme across a variety of eukaryotic taxa. The TR can differ in size, sequence composition, and structure across eukaryotes. This diversity raises an interesting question of how these diverse TR molecules arose. One model used for the acquisition of important TR functional domains originates in the telomerase holoenzyme. This model proposes that when the ancient progenitor linked to modern TRs acquires an internal template region, it necessitates the formation of a physical boundary in the form of the template boundary element (TBE) [15]. This domain is critical as it physically blocks telomerase from utilizing sequences outside the template region during telomere synthesis. This function is key in preventing mutations in the telomere repeat sequence. The synergy between the ancient RT enzyme and the modern TR precursor may be further enhanced by the acquisition of novel structural elements to facilitate RNA–protein interactions. This novel mechanism of regulation and stabilization may promote further interactions and cooperation between the ancient RT enzyme and the modern TR precursor to give rise to the modern telomerase holoenzymes observed today [15]. The TR molecule is processed, and its biogenesis is also highly divergent across a broad range of eukaryotes. In this section, we will discuss the TR processing and biogenesis pathways across representative eukaryotic taxa and provide emerging details of these pathways in deep-branching protists. 

**Figure 1:**
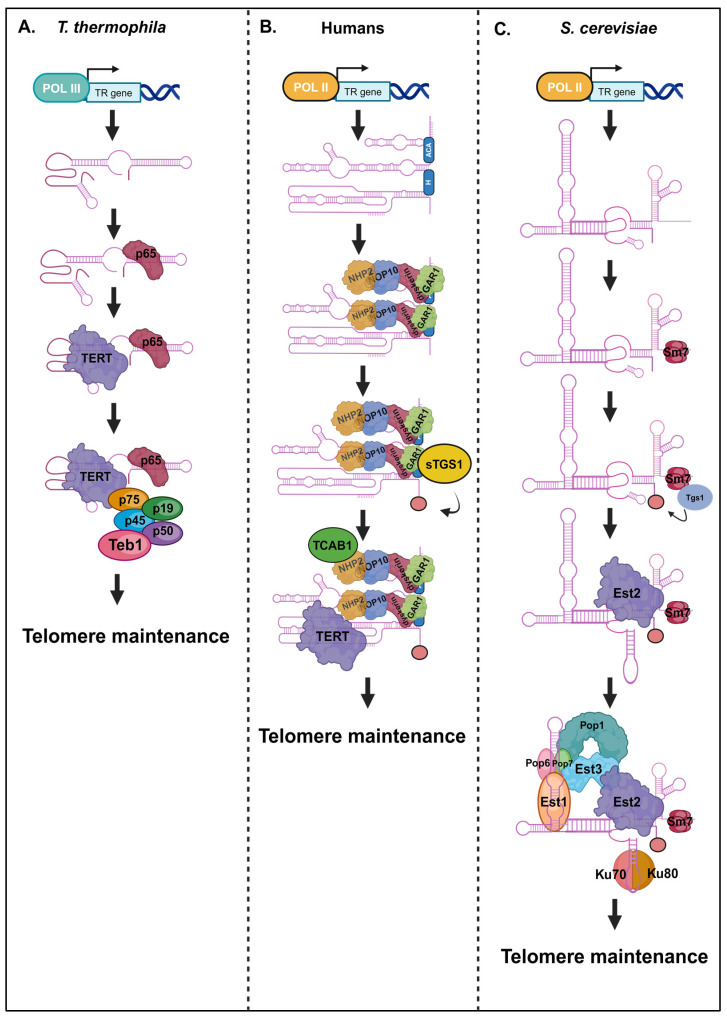
Telomerase RNA processing and biogenesis pathways across representative eukaryotes. (**A**) Telomerase RNA biogenesis in *T. thermophila*. The TR gene is transcribed by RNA polymerase III. The p65 chaperone then binds to fold the TR for TERT interaction purposes. Other accessory proteins then bind to form the catalytically active telomerase holoenzyme. (**B**) Telomerase RNA biogenesis in humans. The TR gene is transcribed by RNA polymerase II. Core H/ACA-box-binding proteins then co-transcriptonally bind to the TR. The sTGS1 protein is recruited to hypermehtylate the 5′ end of the TR to form a TMG cap. TERT and TCAB1 can then bind to the TR to form the active telomerase holoenzyme. (**C**) Telomerase RNA processing in *S. cerevisiae*. The TR is transcribed by RNA polymerase II and the 3′ end is bound and stabilized by the Sm7 complex. This complex Tgs1 is recruited to form the TMG cap on the 5′ end of the RNA. Est2 and other telomerase holoenzyme components then bind to form the complete telomerase complex. The positions of proteins in this model are hypothetical.

An abbreviated pathway for TR processing and biogenesis exists in the free-living ciliate, *T. thermophila* [16]. In these unicellular eukaryotes, the TR gene is transcribed by RNA polymerase III [6]. Other TR molecules in land plants and lower plants have also been shown to be transcribed by RNA POL III, like *T. thermophila* [35,36,68]. This broad conservation in transcription by way of RNA POL III suggests that ciliate and plant TRs share a common origin. This RNA POL III transcription of TR genes was also recently identified in *Hymenoptera* and *Lepidoptera* insects [69]. These recent findings demonstrate the wide expansion of RNA POL-III-mediated transcription factors of TR genes across a diverse set of eukaryotic clades. The acquisition of an RNA POL III biogenesis pathway in diverse eukaryotic clades may have arisen due to convergent evolution. The conservation of similar traits in distantly related organisms may have arisen due to the universal function of telomerase that is essential for telomere maintenance across a variety of eukaryotic organisms. Other eukaryotic TR genes include RNA polymerase II transcripts [70]. Following the transcription of the *T. thermophila* TR gene, the RNA chaperone p65 will bind to and fold the TR in an active conformation for TERT binding [42,43,44]. After the TERT has been associated with the TR, p45, p75, p19, p50, and Teb1 can bind to form the complete telomerase holoenzyme required for telomerase activity and telomere maintenance in these ciliates (Figure 1A). Thus, the proper folding of the TR molecule is critical in order to promote interactions with the TERT protein and it also facilitates the early steps in TERT-TER assembly [71]. Recent studies have also defined the structural and functional requirements of TR and TERT interactions for telomere recruitment and the activation of telomerase in various species. For example, in human telomerase–telomere interactions, shelterin protein TPP1 forms a structured interface with the TERT-specific essential N-terminal elements to promote telomerase activation. This study also uncovered unique TERT–TER interactions that regulate telomerase activity [72]. In fact, methods like the comprehensive identification of RNA-binding proteins via mass spectrometry (ChIRP-MS) are now successfully adopted for plant models to determine TR-interacting partners akin to mammalian telomerase [73].

RNA structures, including TR, are single-stranded molecules that have highly variable and dynamic structures in vivo. The structural dynamics of the TR molecule are relevant to the biogenesis of telomerase holoenzymes, as the TR molecule forms the structural scaffold for accessory protein binding. Traditional RNA structure probing experiments provide still images of RNA structures in vivo with no real insight into in vivo dynamics. Recent studies have begun to highlight the structural dynamics of TR in vivo with possible implications in telomerase complex assembly [74,75]. A recent study found that the ensemble of hTR structures inside the cell consists of a large portion of TR molecules with improperly folded CR4/5 domains that could inhibit accessory protein binding. This highlights the importances of RNA chaperones, like TCAB1, which can bind to and fold the hTR to promote telomerase complex assembly, while other improperly folded forms of the hTR are likely degraded [24,74]. Another recent study in *Physcomitrium patens* (*P. patens*) used in vivo chemical probing complemented by single-molecule structural predictions to highlight the TR dynamics in *P. patens* [75]. The authors identified an ensemble of different TR structures, highlighting the dynamics of the molecule, including an open and closed conformation of the TR. The presence of an active open conformation and an inactive closed conformation of the TR will likely have implications for telomerase catalytic activity and regulation in vivo. The case of whether proteins like RNA chaperones regulate the dynamics between the open and closed conformation of *P. patens* TR remains to be explored, but these structural changes likely help in regulating telomerase catalytic activity and telomerase holoenzyme assembly in vivo.

The TR molecule in the yeasts is rather large, with most being 1000 nt long or more [15]. Despite its large size, much of the *S. cerevisiae* TR sequence was found to be nonessential for telomerase function [76,77]. This suggests that the *S. cerevisiae* TR is rapidly evolving compared to other non-coding RNAs. Zappulla et al. showed that a two-thirds reduction in yeast telomerase RNA can still function in vivo. However, cells with this miniature telomerase RNA (mini T-cells) show selective disadvantages in growth compared to wild-type cells due to their reduced fitness, possibly due to some important non-catalytic functions rendered by TR structural domains. It was also shown that this larger TR molecule is flexible and facilities the interactions of diverse RNA-binding proteins within the telomerase holoenzyme [76]. This model suggests that larger TR molecules were developed to provide flexible structural scaffolds for RNA-binding proteins to facilitate the assembly of a stable telomerase holoenzyme complex. There is a more extensive processing pathway for TR maturation in yeast species that is orchestrated by an ensemble of diverse RNA-binding proteins, which is facilitated by the large structural scaffold provided by the *S. cerevisiae* TR molecule. In *S. cerevisiae*, the TR gene is transcribed by RNA polymerase II, like messenger RNAs (mRNAs). As a consequence of this, the *S. cerevisiae* TR can be found in both polyadenylated and non-polyadenylated forms [78]. It has been shown that the non-polyadenylated form of the TR is associated with the TERT protein [79]. Another consequence of the yeast TR being an RNA polymerase II transcript is the acquisition of a 2,2,7 trimethylguanosine (TMG) cap. The previously mentioned Sm7 complex also binds the TR to facilitate the 5′ capping and 3′ end-processing of the *S. cerevisiae* TR molecule (Figure 1C). Specifically, the Sm7 complex is recruited to TMG synthase, Tgs1, in order to hypermethylate the TMG cap [80,81]. Afterwards, it becomes associated with the TERT, Ku, and Est3 proteins. This 3′ end-processing is unique as yeast TR and is more reliant on RNA splicing factors for its maturation. In the fission yeast, *Schizosaccharomyces pombe* (*S. pombe*), the TR undergoes a unique 3′ end-processing mechanism involving the spliceosomal machinery. Canonical RNA splicing involves two transesterification reactions to remove intronic sequences. 

However, only the first transesterification happens during the end-processing of *S. pombe* TR (*sp*TR) 3′, which results in the removal of the 5′ exon [82]. The *sp*TR also contains an Sm-binding site and is bound by the Sm7 complex to facilitate 5′ and 3′ end-processing events [81]. Following splicing, the 5′ end of *sp*TR is then hypermethylated to form the 5′ TMG cap. Afterwards, the Sm proteins are replaced by the Lsm2-8 complex, which facilitates the *sp*TR association with the TERT and other accessory proteins (like Est1 and Est3) to form the complete telomerase holoenzyme complex [81]. The existence of a 5′ TMG cap in *S. pombe* TR was also highlighted in a separate study where *S. pombe* TR was purified using an anti-TMG antibody. This study also provided a mechanistic explanation of how *S. pombe* telomerase extends a nonuniform telomeric DNA sequence containing rare insertions [83].

The TR in humans is also an RNA polymerase II transcript that undergoes 5′ capping (Figure 1B). The core H/ACA snoRNP-associated proteins co-transcriptionally bind to the TR at the H/ACA box domain of the TR with the help of the H/ACA chaperone, NAF1. This process is further aided by SHQ1, NUFIP, RUVBL1, and RUVBL2 to facilitate H/ACA RNP assembly on the hTR and to facilitate hTR stability and folding. This is followed by hTR 3′ end-processing. The hTR transcript undergoes oligoadenylation via TRF4-2 [84]. These immature forms of the hTR then undergo processing via the poly(A)-specific ribonuclease (PARN) or are degraded by the exosome. These quality control mechanisms may also degrade improperly folded versions of the hTR. The mature hTR transcript is formed following PARN processing [84]. Following 3′ end-processing, NAF1 is then exchanged with the mature H/ACA protein GAR1. The hTR transcript will then undergo 5′ end-processing where the 5′ cap of the hTR molecule is then hypermethylated by the short form of Tgs1 (sTgs1) to form a TMG cap, like yeast TRs [85,86,87]. The mature TR can then become associated with the TERT protein and TCAB1 to form the catalytically active telomerase holoenzyme. 

Less is known about the processing and biogenesis pathways of TR molecules from ancestral eukaryotes. Emerging evidence has indicated the novel features of TR processing and biogenesis in these organisms. In related Kinetoplastid parasites, such as *T. brucei* and *Leishmania*, the TR is transcribed by RNA polymerase II and undergoes *trans*-splicing [45,63]. The TRs of these parasites undergoes *trans*-splicing through the addition of a 5′ spliced leader (SL) cap that replaces the 5′ TMG cap found in humans and yeast. The TRs from these two parasites also appear to contain fractions of the TR molecules that contain a 3′ polyA tail [21,27]. The functional significance of this polyadenylation is unknown in these parasites. A unique feature of kinetoplastid TR molecules is the presence of a C/D snoRNA domain [33,63]. Core C/D-box-binding proteins have been found to be associated with the telomerase complex in *T. brucei* [32,33]. The exact role of these proteins in TR biogenesis and processing is unknown, but they replace the H/ACA box domain found in humans. The presence of a C/D box domain and the association of core C/D box snoRNPs highlight a unique mechanism for TR biogenesis and processing that may be conserved in Kinetoplastid parasites.

An unusual biogenesis pathway that can be used for generating a functional telomerase RNA from a protein-coding mRNA precursor was identified in the fungus *Ustilago maydis* (*U. maydis*). *U. maydis* encodes a roughly 1300 nt long transcript that contains conserved features from other eukaryotic TR molecules, like a CR4/5 domain and a pseudoknot [88]. This gene is transcribed by RNA polymerase II, but then undergoes a unique maturation process that proceeds through a protein-coding mRNA. This maturation likely happens through the endonuclease cleavage of the 3′ UTR of the mRNA precursor to release the mature *U. maydis* TR (*um*TR) [88]. The 3′ end of the *um*TR gene also contains a putative Sm-binding site. This suggests that *um*TR may undergo similar 3′ end-processing to yeast TRs [88]. Because this TR comes from an mRNA precursor, conserved mRNA processing machinery may also facilitate the biogenesis of this RNA through an unidentified mechanism.

### 3.2. Telomerase RNA-Binding Proteins and Telomerase Activity

Among all the TR domains identified across eukaryotes, TR core domain elements and CR4/5 are known to be required for telomerase enzymatic activity. TR catalytic core consists of the template domain that is utilized by the TERT protein for telomeric DNA repeat addition in a species-specific manner. In addition to TERT, telomeric proteins can also become associated with telomerase RNA (TR). For example, the telomerase-associated protein TEP1, a homolog of Tetrahymena telomerase protein p80, specifically interacts with the TERT and the TR, but it is not essential for telomerase activity [89]. In mammals, TRF2 is a telomere-binding protein that interacts with the TR and regulates telomerase activity at chromosome ends. In addition, RNA-binding proteins can also bind to TR and influence telomerase activity. Previously, a telomerase-specific La-related protein of a family of RNA chaperones, P65, was identified in Tetrahymena and was shown to be required for telomerase RNP assembly by binding and inducing structural changes in the TR. NMR and crystal structures have provided evidence [28] that the p65 C-terminal domain interacts with the stem IV of Tetrahymena TR. Further investigations have revealed the role of p65 in stimulating telomerase activity by bridging the interactions between the TR and the TERT [29]. Pof8 is another La family protein identified in fission yeast, *S. pombe*, which can directly interact with the TR molecule. However, unlike p65 in *T. thermophila*, Pof8 recognizes the conserved pseudoknot, a catalytically essential region in the TR, and recruits the Lsm2-8 complex to the 3′ end [90]. In human telomerase, holoenzyme protein TCAB1, which binds to hTR CR4/5, is required for telomerase activity [24].

## 4. Conclusions

Telomerase is the cells’ solution to the end-replication problem. The enzymatic activity of telomerase is critical to maintaining the genomic integrity of a diverse set of eukaryotes. Telomerase is not a singular protein but is composed of many accessory factors that bind together to form the complete telomerase holoenzyme inside cells. A key class of interacting proteins, which are associated with telomerase, are RNA-binding proteins. These proteins are diverse and function in a range of RNA processing and biogenesis pathways. These interactors are often divergent across species owing to differences that arise from distinct TR biogenies and processing pathways in different eukaryotic taxa. The role that these RNA-binding proteins play in telomerase regulation and TR processing have been well studied in the context of *Tetrahymena*, human and yeast, all of which are model eukaryotes. Telomerase RNA-binding proteins can bind to the RNA and stabilize it, preventing its degradation and ensuring its availability for telomerase assembly and activity.

The role of RNA-binding proteins in telomerase regulation in protozoan parasites is less well understood. These include clinically relevant parasites like *T. brucei* that cause human disease. Recent studies have begun to illuminate the roles of RNA-binding proteins in telomerase regulation in *T. brucei*. The work conducted so far has highlighted the presence of novel regulatory pathways of telomere maintenance in these organisms. This is significant as *T. brucei* is dependent on constant telomerase activity for its proliferation and virulence. Further studies are needed to help us understand the unique telomerase regulatory mechanisms that exist in *T. brucei*.

In general, it appears that telomerase RNA-binding proteins have likely undergone adaptations to accommodate the specific telomere biology of different organisms, including differences in telomere length regulation, telomerase recruitment mechanisms, and telomerase subunit composition. Comparative studies of telomerase and telomerase RNA-binding proteins across diverse species can provide insights into the evolutionary dynamics of telomere maintenance pathways and shed light on the functional significance of telomerase-associated proteins.

## Figures and Tables

**Table 1:** RNA-binding proteins that interact with the telomerase holoenzyme. Functions of selected proteins are discussed in the text.

Protein	Species	Accession Number (Uniprot)	References
Dyskerin	Human	O60832	[19,22]
NHP2	Human	Q9NX24	[22]
NOP10	Human	Q9NPE3	[22]
GAR1	Human	Q9NY12	[22]
TCAB1	Human	Q9BUR4	[21,24]
Est1	*S. cerevisiae*	P17214	[25]
Pop1	*S. cerevisiae*	P41812	[26]
Pop6	*S. cerevisiae*	P53218	[26]
Pop7	*S. cerevisiae*	P38291	[26]
Smd2	*S. cerevisiae*	Q06217	[25]
Smb1	*S. cerevisiae*	P40018	[25]
Smd3	*S. cerevisiae*	P43321	[25]
p65	*T. thermophila*	W7X6T2	[27,28,29,30,31]
p75	*T. thermophila*	A0PGB2	[27,31]
p45	*T. thermophila*	Q6JXI5	[27,31]
p19	*T. thermophila*	D2CVN7	[27,31]
p50	*T. thermophila*	D2CVN8	[27,31]
NOP56	*T. brucei*	Q580Z5	[32,33]
NOP58	*T. brucei*	Q38F23	[32,33]
Fibrillarin (NOP1)	*T. brucei*	Q38AL4	[32,33]

## Data Availability

No new data was created or analyzed in this study. Data sharing is not applicable to this article.

## References

[1] De Lange T. (2009). How telomeres solve the end-protection problem. Science.

[2] De Lange T. (2018). Shelterin-mediated telomere protection. Annu. Rev. Genet..

[3] Price C., Boltz K.A., Chaiken M.F., Stewart J.A., Beilstein M.A., Shippen D.E. (2010). Evolution of CST function in telomere maintenance. Cell Cycle.

[4] Lingner J., Cooper J.P., Cech T.R. (1995). Telomerase and DNA end replication: No longer a lagging strand problem?. Science.

[5] Kong C.M., Lee X.W., Wang X. (2013). Telomere shortening in human diseases. FEBS J..

[6] Greider C.W., Blackburn E.H. (1989). A telomeric sequence in the RNA of Tetrahymena telomerase required for telomere repeat synthesis. Nature.

[7] Autexier C., Pruzan R., Funk W.D., Greider C.W. (1996). Reconstitution of human telomerase activity and identification of a minimal functional region of the human telomerase RNA. EMBO J..

[8] Weinrich S.L., Pruzan R., Ma L., Ouellette M., Tesmer V.M., Holt S.E., Bodnar A.G., Lichtsteiner S., Kim N.W., Trager J.B. (1997). Reconstitution of human telomerase with the template RNA component hTR and the catalytic protein subunit hTRT. Nat. Genet..

[9] Collins K. (2006). The biogenesis and regulation of telomerase holoenzymes. Nat. Rev. Mol. Cell Biol..

[10] Davis J.A., Chakrabarti K. (2022). Telomerase ribonucleoprotein and genome integrity—An emerging connection in protozoan parasites. Wiley Interdiscip. Rev. RNA.

[11] Moriarty T.J., Huard S., Dupuis S., Autexier C. (2002). Functional multimerization of human telomerase requires an RNA interaction domain in the N terminus of the catalytic subunit. Mol. Cell. Biol..

[12] Bley C.J., Qi X., Rand D.P., Borges C.R., Nelson R.W., Chen J.J.-L. (2011). RNA–protein binding interface in the telomerase ribonucleoprotein. Proc. Natl. Acad. Sci. USA.

[13] Huang J., Brown A.F., Wu J., Xue J., Bley C.J., Rand D.P., Wu L., Zhang R., Chen J.J., Lei M. (2014). Structural basis for protein-RNA recognition in telomerase. Nat. Struct. Mol. Biol..

[14] Jansson L.I., Akiyama B.M., Ooms A., Lu C., Rubin S.M., Stone M.D. (2015). Structural basis of template-boundary definition in Tetrahymena telomerase. Nat. Struct. Mol. Biol..

[15] Podlevsky J.D., Chen J.J.-L. (2016). Evolutionary perspectives of telomerase RNA structure and function. RNA Biol..

[16] Egan E.D., Collins K. (2012). Biogenesis of telomerase ribonucleoproteins. RNA.

[17] Nakamura T.M., Cech T.R. (1998). Reversing time: Origin of telomerase. Cell.

[18] De Lange T. (2015). A loopy view of telomere evolution. Front. Genet..

[19] Cohen S.B., Graham M.E., Lovrecz G.O., Bache N., Robinson P.J., Reddel R.R. (2007). Protein composition of catalytically active human telomerase from immortal cells. Science.

[20] Fu D., Collins K. (2007). Purification of human telomerase complexes identifies factors involved in telomerase biogenesis and telomere length regulation. Mol. Cell.

[21] Venteicher A.S., Abreu E.B., Meng Z., McCann K.E., Terns R.M., Veenstra T.D., Terns M.P., Artandi S.E. (2009). A human telomerase holoenzyme protein required for Cajal body localization and telomere synthesis. Science.

[22] Nguyen T.H.D., Tam J., Wu R.A., Greber B.J., Toso D., Nogales E., Collins K. (2018). Cryo-EM structure of substrate-bound human telomerase holoenzyme. Nature.

[23] Ghanim G.E., Fountain A.J., Van Roon A.-M.M., Rangan R., Das R., Collins K., Nguyen T.H.D. (2021). Structure of human telomerase holoenzyme with bound telomeric DNA. Nature.

[24] Chen L., Roake C.M., Freund A., Batista P.J., Tian S., Yin Y.A., Gajera C.R., Lin S., Lee B., Pech M.F. (2018). An activity switch in human telomerase based on RNA conformation and shaped by TCAB1. Cell.

[25] Lin K.W., McDonald K.R., Guise A.J., Chan A., Cristea I.M., Zakian V.A. (2015). Proteomics of yeast telomerase identified Cdc48-Npl4-Ufd1 and Ufd4 as regulators of Est1 and telomere length. Nat. Commun..

[26] Lemieux B., Laterreur N., Perederina A., Noël J.-F., Dubois M.-L., Krasilnikov A.S., Wellinger R.J. (2016). Active yeast telomerase shares subunits with ribonucleoproteins RNase P and RNase MRP. Cell.

[27] Jiang J., Chan H., Cash D.D., Miracco E.J., Ogorzalek Loo R.R., Upton H.E., Cascio D., O’Brien Johnson R., Collins K., Loo J.A. (2015). Structure of Tetrahymena telomerase reveals previously unknown subunits, functions, and interactions. Science.

[28] Singh M., Wang Z., Koo B.-K., Patel A., Cascio D., Collins K., Feigon J. (2012). Structural basis for telomerase RNA recognition and RNP assembly by the holoenzyme La family protein p65. Mol. Cell.

[29] Berman A.J., Gooding A.R., Cech T.R. (2010). Tetrahymena Telomerase Protein p65 Induces Conformational Changes throughout Telomerase RNA (TER) and Rescues Telomerase Reverse Transcriptase and TER Assembly Mutants. Mol. Cell. Biol..

[30] Wang Y., He Y., Wang Y., Yang Y., Singh M., Eichhorn C.D., Cheng X., Jiang Y.X., Zhou Z.H., Feigon J. (2023). Structure of LARP7 Protein p65–telomerase RNA Complex in Telomerase Revealed by Cryo-EM and NMR. J. Mol. Biol..

[31] Jiang J., Miracco E.J., Hong K., Eckert B., Chan H., Cash D.D., Min B., Zhou Z.H., Collins K., Feigon J. (2013). The architecture of Tetrahymena telomerase holoenzyme. Nature.

[32] Davis J.A., Reyes A.V., Saha A., Wolfgeher D.J., Xu S.-L., Truman A.W., Li B., Chakrabarti K. (2023). Proteomic analysis defines the interactome of telomerase in the protozoan parasite, Trypanosoma brucei. Front. Cell Dev. Biol..

[33] Gupta S.K., Kolet L., Doniger T., Biswas V.K., Unger R., Tzfati Y., Michaeli S. (2013). The Trypanosoma brucei telomerase RNA (TER) homologue binds core proteins of the C/D snoRNA family. FEBS Lett..

[34] Nguyen T.H.D., Collins K., Nogales E. (2019). Telomerase structures and regulation: Shedding light on the chromosome end. Curr. Opin. Struct. Biol..

[35] Song J., Logeswaran D., Castillo-González C., Li Y., Bose S., Aklilu B.B., Ma Z., Polkhovskiy A., Chen J.J.-L., Shippen D.E. (2019). The conserved structure of plant telomerase RNA provides the missing link for an evolutionary pathway from ciliates to humans. Proc. Natl. Acad. Sci. USA.

[36] Fajkus P., Peška V., Závodník M., Fojtová M., Fulnečková J., Dobias Š., Kilar A., Dvořáčková M., Zachová D., Nečasová I. (2019). Telomerase RNAs in land plants. Nucleic Acids Res..

[37] Procházková Schrumpfová P., Schořová Š., Fajkus J. (2016). Telomere-and telomerase-associated proteins and their functions in the plant cell. Front. Plant Sci..

[38] Schrumpfova P.P., Vychodilova I., Dvořáčková M., Majerska J., Dokladal L., Schořová Š., Fajkus J. (2014). Telomere repeat binding proteins are functional components of Arabidopsis telomeres and interact with telomerase. Plant J..

[39] Kusová A., Steinbachová L., Přerovská T., Drábková L.Z., Paleček J., Khan A., Rigóová G., Gadiou Z., Jourdain C., Stricker T. (2023). Completing the TRB family: Newly characterized members show ancient evolutionary origins and distinct localization, yet similar interactions. Plant Mol. Biol..

[40] Wang Y., Sušac L., Feigon J. (2019). Structural biology of telomerase. Cold Spring Harb. Perspect. Biol..

[41] Shakirov E.V., Surovtseva Y.V., Osbun N., Shippen D.E. (2005). The Arabidopsis Pot1 and Pot2 proteins function in telomere length homeostasis and chromosome end protection. Mol. Cell. Biol..

[42] Song J., Castillo-González C., Ma Z., Shippen D.E. (2021). Arabidopsis retains vertebrate-type telomerase accessory proteins via a plant-specific assembly. Nucleic Acids Res..

[43] Kannan K., Nelson A.D., Shippen D.E. (2008). Dyskerin is a component of the Arabidopsis telomerase RNP required for telomere maintenance. Mol. Cell. Biol..

[44] Min B., Collins K. (2009). An RPA-related sequence-specific DNA-binding subunit of telomerase holoenzyme is required for elongation processivity and telomere maintenance. Mol. Cell.

[45] Sandhu R., Sanford S., Basu S., Park M., Pandya U.M., Li B., Chakrabarti K. (2013). A trans-spliced telomerase RNA dictates telomere synthesis in Trypanosoma brucei. Cell Res..

[46] Dey A., Monroy-Eklund A., Klotz K., Saha A., Davis J., Li B., Laederach A., Chakrabarti K. (2021). In vivo architecture of the telomerase RNA catalytic core in Trypanosoma brucei. Nucleic Acids Res..

[47] Nittis T., Guittat L., LeDuc R.D., Dao B., Duxin J.P., Rohrs H., Townsend R.R., Stewart S.A. (2010). Revealing novel telomere proteins using in vivo cross-linking, tandem affinity purification, and label-free quantitative LC-FTICR-MS. Mol Cell Proteom..

[48] Sharma N.K., Reyes A., Green P., Caron M.J., Bonini M.G., Gordon D.M., Holt I.J., Santos J.H. (2012). Human telomerase acts as a hTR-independent reverse transcriptase in mitochondria. Nucleic Acids Res..

[49] Takenaka Y., Matsuura T., Haga N., Mitsui Y. (2001). Expression of telomerase reverse transcriptase and telomere elongation during sexual maturation in Paramecium caudatum. Gene.

[50] Amanda J.Y., Romero D.P. (2002). A unique pause pattern during telomere addition by the error-prone telomerase from the ciliate Paramecium tetraurelia. Gene.

[51] Figueiredo L.M., Rocha E.P., Mancio-Silva L., Prevost C., Hernandez-Verdun D., Scherf A. (2005). The unusually large Plasmodium telomerase reverse-transcriptase localizes in a discrete compartment associated with the nucleolus. Nucleic Acids Res..

[52] Giardini M.A., Lira C.B., Conte F.F., Camillo L.R., de Siqueira Neto J.L., Ramos C.H., Cano M.I.N. (2006). The putative telomerase reverse transcriptase component of Leishmania amazonensis: Gene cloning and characterization. Parasitol. Res..

[53] Malik H.S., Burke W.D., Eickbush T.H. (2000). Putative telomerase catalytic subunits from Giardia lamblia and Caenorhabditis elegans. Gene.

[54] Nassir N., Hyde G.J., Baskar R. (2019). A telomerase with novel non-canonical roles: TERT controls cellular aggregation and tissue size in Dictyostelium. PLoS Genet..

[55] Rajkowitsch L., Chen D., Stampfl S., Semrad K., Waldsich C., Mayer O., Jantsch M.F., Konrat R., Bläsi U., Schroeder R. (2007). RNA chaperones, RNA annealers and RNA helicases. RNA Biol..

[56] Stern J.L., Zyner K.G., Pickett H.A., Cohen S.B., Bryan T.M. (2012). Telomerase recruitment requires both TCAB1 and Cajal bodies independently. Mol. Cell. Biol..

[57] Venteicher A.S., Artandi S.E. (2009). TCAB1: Driving telomerase to Cajal bodies. Cell Cycle.

[58] Vasianovich Y., Bajon E., Wellinger R.J. (2020). Telomerase biogenesis requires a novel Mex67 function and a cytoplasmic association with the Sm7 complex. Elife.

[59] Witkin K.L., Collins K. (2004). Holoenzyme proteins required for the physiological assembly and activity of telomerase. Genes Dev..

[60] Dock-Bregeon A.-C., Lewis K.A., Conte M.R. (2021). The La-related proteins: Structures and interactions of a versatile superfamily of RNA-binding proteins. RNA Biol..

[61] Fu D., Collins K. (2003). Distinct biogenesis pathways for human telomerase RNA and H/ACA small nucleolar RNAs. Mol. Cell.

[62] Egan E.D., Collins K. (2012). An enhanced H/ACA RNP assembly mechanism for human telomerase RNA. Mol. Cell. Biol..

[63] Vasconcelos E.J., Nunes V.S., da Silva M.S., Segatto M., Myler P.J., Cano M.I.N. (2014). The putative Leishmania telomerase RNA (Leish TER) undergoes trans-splicing and contains a conserved template sequence. PloS ONE.

[64] Podlevsky J.D., Bley C.J., Omana R.V., Qi X., Chen J.J.-L. (2007). The telomerase database. Nucleic Acids Res..

[65] Bosch J.A., Chen C.L., Perrimon N. (2021). Proximity-dependent labeling methods for proteomic profiling in living cells: An update. Wiley Interdiscip. Rev. Dev. Biol..

[66] Han S., Zhao B.S., Myers S.A., Carr S.A., He C., Ting A.Y. (2020). RNA–protein interaction mapping via MS2-or Cas13-based APEX targeting. Proc. Natl. Acad. Sci. USA.

[67] Ivanyi-Nagy R., Ahmed S.M., Peter S., Ramani P.D., Ong P.F., Dreesen O., Dröge P. (2018). The RNA interactome of human telomerase RNA reveals a coding-independent role for a histone mRNA in telomere homeostasis. Elife.

[68] Fajkus P., Kilar A., Nelson A.D., Holá M., Peška V., Goffová I., Fojtová M., Zachová D., Fulnečková J., Fajkus J. (2021). Evolution of plant telomerase RNAs: Farther to the past, deeper to the roots. Nucleic Acids Res..

[69] Fajkus P., Adámik M., Nelson A.D., Kilar A.M., Franek M., Bubeník M., Frydrychová R.Č., Votavová A., Sýkorová E., Fajkus J. (2023). Telomerase RNA in Hymenoptera (Insecta) switched to plant/ciliate-like biogenesis. Nucleic Acids Res..

[70] Logeswaran D., Li Y., Podlevsky J.D., Chen J.J.-L. (2021). Monophyletic origin and divergent evolution of animal telomerase RNA. Mol. Biol. Evol..

[71] Cash D.D., Feigon J. (2017). Structure and folding of the Tetrahymena telomerase RNA pseudoknot. Nucleic Acids Res..

[72] Liu B., He Y., Wang Y., Song H., Zhou Z.H., Feigon J. (2022). Structure of active human telomerase with telomere shelterin protein TPP1. Nature.

[73] Bozdechova L., Rudolfova A., Hanakova K., Fojtova M., Fajkus J. (2024). Optimizing ChIRP-MS for Comprehensive Profiling of RNA-Protein Interactions in Arabidopsis thaliana: A Telomerase RNA Case Study. Plants.

[74] Forino N.M., Woo J.Z., Zaug A.J., Jimenez A.G., Cech T.R., Rouskin S., Stone M.D. (2023). Dissecting telomerase RNA structural heterogeneity in living human cells with DMS-MaPseq. bioRxiv.

[75] Bozděchová L., Havlová K., Fajkus P., Fajkus J. (2024). Analysis of Telomerase RNA Structure in Physcomitrium patens Indicates Functionally Relevant Transitions Between OPEN and CLOSED Conformations. J. Mol. Biol..

[76] Zappulla D.C., Cech T.R. (2004). Yeast telomerase RNA: A flexible scaffold for protein subunits. Proc. Natl. Acad. Sci. USA.

[77] Zappulla D.C. (2020). Yeast telomerase RNA flexibly scaffolds protein subunits: Results and repercussions. Molecules.

[78] Chapon C., Cech T., Zaug A. (1997). Polyadenylation of telomerase RNA in budding yeast. RNA.

[79] Bosoy D., Lue N.F. (2004). Yeast telomerase is capable of limited repeat addition processivity. Nucleic Acids Res..

[80] Seto A.G., Zaug A.J., Sobel S.G., Wolin S.L., Cech T.R. (1999). Saccharomyces cerevisiae telomerase is an Sm small nuclear ribonucleoprotein particle. Nature.

[81] Tang W., Kannan R., Blanchette M., Baumann P. (2012). Telomerase RNA biogenesis involves sequential binding by Sm and Lsm complexes. Nature.

[82] Box J.A., Bunch J.T., Tang W., Baumann P. (2008). Spliceosomal cleavage generates the 3′ end of telomerase RNA. Nature.

[83] Webb C.J., Zakian V.A. (2008). Identification and characterization of the Schizosaccharomyces pombe TER1 telomerase RNA. Nat. Struct. Mol. Biol..

[84] Tseng C.-K., Wang H.-F., Burns A.M., Schroeder M.R., Gaspari M., Baumann P. (2015). Human telomerase RNA processing and quality control. Cell Rep..

[85] Jády B.E., Bertrand E., Kiss T. (2004). Human telomerase RNA and box H/ACA scaRNAs share a common Cajal body–specific localization signal. J. Cell Biol..

[86] Fu D., Collins K. (2006). Human telomerase and Cajal body ribonucleoproteins share a unique specificity of Sm protein association. Genes Dev..

[87] Girard C., Verheggen C., Neel H., Cammas A., Vagner S., Soret J., Bertrand E., Bordonne R. (2008). Characterization of a short isoform of human Tgs1 hypermethylase associating with small nucleolar ribonucleoprotein core proteins and produced by limited proteolytic processing. J. Biol. Chem..

[88] Logeswaran D., Li Y., Akhter K., Podlevsky J.D., Olson T.L., Forsberg K., Chen J.J.-L. (2022). Biogenesis of telomerase RNA from a protein-coding mRNA precursor. Proc. Natl. Acad. Sci. USA.

[89] Liu Y., Snow B.E., Hande M.P., Baerlocher G., Kickhoefer V.A., Yeung D., Wakeham A., Itie A., Siderovski D.P., Lansdorp P.M. (2000). Telomerase-associated protein TEP1 is not essential for telomerase activity or telomere length maintenance in vivo. Mol. Cell. Biol..

[90] Hu X., Kim J.K., Yu C., Jun H.I., Liu J., Sankaran B., Huang L., Qiao F. (2020). Quality-Control Mechanism for Telomerase RNA Folding in the Cell. Cell Rep..

